# State Derivation of a 12-Axis Gyroscope-Free Inertial Measurement Unit

**DOI:** 10.3390/s110303145

**Published:** 2011-03-14

**Authors:** Jau-Ching Lu, Pei-Chun Lin

**Affiliations:** Department of Mechanical Engineering, National Taiwan University, No.1 Roosevelt Rd. Sec.4, ME Eng. Bldg. Room 503-3, Taipei, Taiwan; E-Mail: b93502114@ntu.edu.tw

**Keywords:** inertial measurement unit, accelerometer, gyroscope-free, angular velocity, interacting multiple model, context-based

## Abstract

The derivation of linear acceleration, angular acceleration, and angular velocity states from a 12-axis gyroscope-free inertial measurement unit that utilizes four 3-axis accelerometer measurements at four distinct locations is reported. Particularly, a new algorithm which derives the angular velocity from its quadratic form and derivative form based on the context-based interacting multiple model is demonstrated. The performance of the system was evaluated under arbitrary 3-dimensional motion.

## 1. Introduction

Inertial sensors have been widely used in various applications, including motion detection [1], body state estimation [2–4], navigation [5–7], microsurgery [8], rehabilitation [9], *etc.* Traditionally a standard inertial measurement unit (IMU) comprised of 3-axis linear acceleration measurement by accelerometers installed at center of mass (COM) and 3-axis angular velocity measurement by rate gyros readily provides complete six degree-of-freedom (DOF) motion-related measurements spanning the 3-dimensional space. For highly dynamic systems which favorably have angular acceleration measurements, to the best of our knowledge there is no off-the-shelf product available. One widely adopted approach to derive this state is by differentiation of rate gyro signals, together with the filter technique. The other approach is based on the principle of Newtonian Mechanics, which relates linear acceleration, angular acceleration, and angular velocity in a memoryless manner. Because of this characteristic, derivation of the angular acceleration by only the inertial sensors seems to be a feasible method [10].

The gyroscope-free inertial measurement unit (GF-IMU) [11–13] is one of the more popular IMU methods to achieve this goal. Compared to the traditional IMU, the GF-IMU utilizing only accelerometers includes several features such as low-cost, easy calibration, being less affected by temperature variations, and a simple mechatronic setup. In general, the GF-IMU is capable of deriving linear acceleration, angular acceleration, and angular velocity. Because the latter two states have integrative/derivative relation, a GF-IMU comprised of 6-axis measurements is theoretically capable of yielding all three states (*i.e.*, 9 scalar unknowns). One of the typical configurations of sensors is to have a 3-axis acceleration measurement at the COM and three 1-axis measurements on the principal axes. However, the iterative computation between the derived angular acceleration and the integrated angular velocity can possibly deteriorate the fidelity of these two states. Padgaonkar *et al.* proposed a 9-axis acceleration measurement system capable of deriving bounded linear and angular acceleration [14]. Chen *et al.* proposed a novel 6-axis system which yielded a bounded angular acceleration [15]. The system was carefully evaluated [16] and improved by adding a 3-axis acceleration measurement [17]. In general, due to the quadratic formulation of angular velocity in the rigid body dynamics, the derivation of this state in the 9-axis IMU faces the sign ambiguity problem [18]. This dilemma can be solved by comparing it to the estimated angular velocity which is integrated from the angular acceleration measurement [19] or by adding the redundant measurements to the IMU, for example, to increase the measurements to 12-axis [20]. Parsa *et al.* later developed an original all-accelerometer IMU which requires twelve 1-axis accelerometers mounted at specific locations on the surfaces of a cube. The system is capable of deriving all three states in which the angular velocity was derived through an optimization procedure from six measured inputs in the quadratic form [21]. Schopp *et al.* reported another novel 12-axis IMU which was constructed by twelve 1-axis accelerometers in different configurations and utilized an Unscented Kalman Filter (UKF) to yield all three states simultaneously [22].

Previously we had installed a 12-axis IMU composed of four 3-axis accelerometers at four distinct locations on the robot RHex [23], together with some custom-made leg sensors [24], to perform sensor data fusion for full body state estimation in this hexapod robot with dynamical gait [25]. Based on the rigid-body dynamics and matrix theory, the developed 12-axis IMU is theoretically capable of deriving all three states. However, limited available space on the RHex for sensor installation at that time constrained the configuration of the IMU far from the optimum level. Only the linear and angular accelerations were available for further analysis and no angular velocity developments were performed.

Here, we report on the state derivation and performance evaluation of the 12-axis IMU with optimal configuration in the sense of matrix operation, allowing the system to yield all three states. Particularly, a new algorithm which derives the angular velocity is reported. Basically, the state is estimated by the mixed signals from its quadratic form and derivative form based on the context-based interacting multiple models (IMM) [26]. The algorithm requires low computation power suitable for real-time derivation of the state. The proposed 12-axis IMU in its new configuration was tested under 3-dimensional random motion with various magnitudes, and its performance was evaluated by comparing to the results from the traditional IMU installed at the COM.

Section 2 briefly reviews the construction of the 12-axis IMU based on the analysis of rigid body dynamics. Section 3 describes the derivation of the angular velocity by the context-based IMM in detail. Section 4 reports the results of experimental evaluation, and Section 5 concludes the work.

## 2. Construction of the 12-Axis IMU

**Figure 1.**
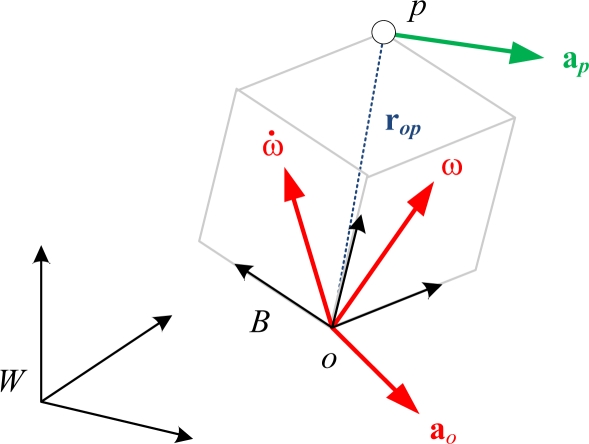
General description of the accelerated body in the inertial frame.

A brief review regarding construction of the 12-axis IMU is described in this section [25]. As shown in Figure 1, the acceleration vector, **a***_p_*, of a point, *p*, rigidly attached to an accelerating body frame *B* with origin *o*, in the inertial frame, *W*, is a function of the body’s angular velocity, **ω**, angular acceleration, **ω̇**, and translational acceleration of the body origin, **a***_o_*, represented by:

(1) $$a_{p}=a_{o}+\dot{\omega }\times r_{\mathrm{op}}+\omega \times (\omega \times r_{\mathrm{op}})$$

where **r***_op_*, the fixed position vector of point *p* relative to *o*, is assumed to be known. In general, the three body states (*i.e.*, 9 scalar values) on the right hand side of Equation (1) are unknowns, including the COM translational acceleration, **a***_COM_* (usually equal to the origin of body frame, **a***_o_*), the body angular acceleration, and the angular velocity:

(2) $$\begin{array}{l} a_{o}=a_{\mathrm{COM}}={\left[\begin{array}{ccc} a_{x} & a_{y} & a_{z} \end{array}\right]}^{T}\\ \dot{\omega }={\left[\begin{array}{ccc} {\dot{\omega }}_{x} & {\dot{\omega }}_{y} & {\dot{\omega }}_{z} \end{array}\right]}^{T}\\ \omega ={\left[\begin{array}{ccc} \omega_{x} & \omega_{y} & \omega_{z} \end{array}\right]}^{T} \end{array}$$

With the quadratic representation of the angular velocity:

(3) $$\omega_{6}(\omega )={\left[\begin{array}{llllll} \omega_{x}^{2}+\omega_{y}^{2} & \omega_{x}^{2}+\omega_{z}^{2} & \omega_{y}^{2}+\omega_{z}^{2} & \omega_{x}\omega_{y} & \omega_{x}\omega_{z} & \omega_{y}\omega_{z} \end{array}\right]}^{T}$$

Equation (1) appears to be linear with these 12 scalar unknowns:

(4) $$\begin{array}{ll} x_{\text{var}} & ={\left[\begin{array}{ccc} a_{o}^{T} & {\dot{\omega }}^{T} & \omega_{6}{\left(\omega \right)}^{T} \end{array}\right]}^{T}\\ & ={\left[\begin{array}{llllllllllll} a_{x} & a_{y} & a_{z} & {\dot{\omega }}_{x} & {\dot{\omega }}_{y} & {\dot{\omega }}_{z} & \omega_{x}^{2}+\omega_{y}^{2} & \omega_{x}^{2}+\omega_{z}^{2} & \omega_{y}^{2}+\omega_{z}^{2} & \omega_{x}\omega_{y} & \omega_{x}\omega_{z} & \omega_{y}\omega_{z} \end{array}\right]}^{T} \end{array}$$

Presumably four 3-axis accelerometers are installed at point *p_j, j=1,2,3,4_* with known **r***_opj, j=1,2,3,4_* :

$$r_{m}={\left[\begin{array}{cccc} r_{\mathrm{op}1}^{T} & r_{\mathrm{op}2}^{T} & r_{\mathrm{op}3}^{T} & r_{\mathrm{op}4}^{T} \end{array}\right]}^{T}\text{with}\;r_{\mathrm{opj}}={\left[\begin{array}{ccc} r_{\mathrm{opjx}} & r_{\mathrm{opjy}} & r_{\mathrm{opjz}} \end{array}\right]}^{T}\;\;\;j=1,2,3,4$$

and these accelerometers are oriented to measure accelerations in the directions along with three principal axes of the body coordinate, **a***_pj, j=1∼4_*:

(5) $$a_{m}={\left[\begin{array}{cccc} a_{p1}^{T} & a_{p2}^{T} & a_{p3}^{T} & a_{p4}^{T} \end{array}\right]}^{T}\text{with}\;a_{\mathrm{pj}}={\left[\begin{array}{ccc} a_{\mathrm{pjx}} & a_{\mathrm{pjy}} & a_{\mathrm{pjz}} \end{array}\right]}^{T}\;\;\;j=1,2,3,4$$

a linear system with twelve equations and twelve unknowns is formed:

(6) $$\begin{array}{ll} a_{m} & =S\left(r_{m}\right)x_{\text{var}}\\ & =\left[\begin{array}{cccccccccccc} 1 & 0 & 0 & 0 & r_{1z} & -r_{1y} & 0 & 0 & -r_{1x} & r_{1y} & r_{1z} & 0\\ 0 & 1 & 0 & -r_{1z} & 0 & r_{1x} & 0 & -r_{1y} & 0 & r_{1x} & 0 & r_{1z}\\ 0 & 0 & 1 & r_{1y} & -r_{1x} & 0 & -r_{1z} & 0 & 0 & 0 & r_{1x} & r_{1y}\\ \vdots & \vdots & \vdots & \vdots & \vdots & \vdots & \vdots & \vdots & \vdots & \vdots & \vdots & \vdots \end{array}\right] \end{array}x_{\text{var}}$$

where **S**(**r_m_**) is the 12 × 12 matrix and hereafter referred to as the “structure matrix”. The **S**(**r_m_**) is the combination of four copies of Equation (1) with the dimensions 3 × 12. Due to the similarity of motion along with three principal axes, the structure of the 3 × 12 matrix is symmetric at a certain level. The first 3 × 3 matrix from the left side of **S**(**r_m_**) is just an identity matrix and the second 3 × 3 matrix from the left side is the skew-symmetric matrix because of the cross product operator. The 3 × 6 matrix from the right side of **S**(**r_m_**) is generated by the double cross product of the angular velocity term.

The unknown body states can now be derived by the matrix operation:

(7) $$x_{\text{var}}=S{\left(r_{m}\right)}^{-1}a_{m}$$

**Figure 2.**
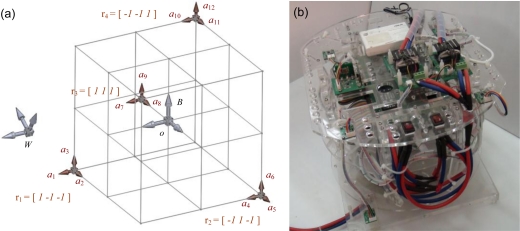
(**a**) The configuration of four 3-axis accelerometers that yields the best condition number for structure matrix **S**(**r_m_**). (**b**) The experimental apparatus.

Equation (7) reveals that the extraction of the desired state, **x**_var_, now depends on the rank and numerical condition of the “structure matrix”, **S**(**r_m_**), which is solely a function of the positions of accelerometers, **r_m_**. Previously the numerical exploration pointed out that allocation of the four sensors shown in Figure 2(a) yields the best condition number of **S**(**r_m_**), square root of 2. It indicates that this configuration is the most appropriate for matrix inversion [25], and the computation error induced by the matrix inversion is small. Therefore the new experimental setup shown in Figure 2(b) was built according to this configuration for the following analysis. Please note that the construction and inversion of the structure matrix **S**(**r_m_**) only needs to be done once and can be computed offline after the positions and orientations of the accelerometers are determined.

## 3. Derivation of the Angular Velocity from the 12-Axis GF-IMU

Section 2 shows that in the real-time process the unknown body state **x**_var_ in the proposed 12-axis GF-IMU can be derived from the 12 sensed scalar accelerations multiplied with the inversed structure matrix shown in Equation (7). In **x**_var_, the linear and angular accelerations are readily derived though the angular velocity in its exact form is still unsolved and requires further computation. In the current formulation, two sensed sources are available for this computation. One is from the angular acceleration, the 4th–6th terms of **x**_var_ shown in Equation (4), which is the derivative of the desired state. The other is from the last six terms of **x**_var_, which is the quadratic form of the desired one. Because in the empirical setup the developed algorithm is executed by the commercial computers, the representation is in the discrete domain in the following sections.

### 3.1. The Primitive Derivation of the Angular Velocity

To derive the angular velocity from the available angular acceleration, **ω***_int_*, the trapezoid integration is the preferred method:

(8) $$\omega_{\text{int}}:\;\;\omega_{i,j}=\omega_{i,j-1}+\Delta t(\frac{{\dot{\omega }}_{i,j-1}+{\dot{\omega }}_{i,j}}{2}),i=x,y,z$$

where the subscripts *j* and *j−1* represent consecutive two time stamps with time, Δ*t*, in between. This method (hereafter referred to as the “integration method”) is simple and effective for a short duration, but is not suitable for a long period of time because the accumulated integration error quickly deteriorates the quality of the derived signal. Adding a bias term to Equation (8) may reduce the error; however, in general this compensation is not effective for motion not performed in specific patterns.

**Figure 3.**
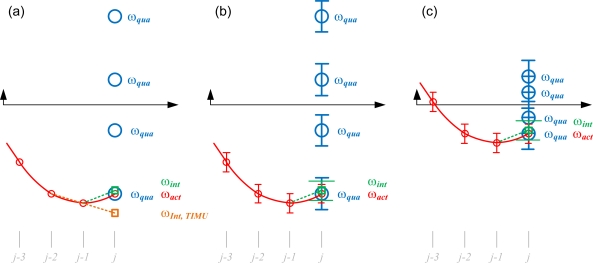
Various scenarios of selecting the correct angular velocity from the eight candidates deriving from the quadratic method: (**a**) “perfect world” with no estimate error, the true angular velocity (red line) is precisely sampled (red circle). The initial guess derived from integration method is represented in green color. (**b**) Normal situation where all computed data have certain variances (shown in error bar). (**c**) Normal situation when the magnitude of the angular velocity is small and the correct solution is hard to pick.

The angular velocity derived from its quadratic form (hereafter referred to as the “quadratic method”) has non-drift nature; however, the trade-off is the sign ambiguity problem, meaning to select the correct answer from multiple potential choices resulted from “square-root” computations. More specifically, assuming the first three quadratic terms shown in Equation (3) are chosen:

(9) $$\begin{array}{ccc} \omega_{x}^{2}+\omega_{y}^{2}=a & \omega_{x}^{2}+\omega_{z}^{2}=b & \omega_{y}^{2}+\omega_{z}^{2}=c \end{array}$$

a solution derived from this method, **ω***_qua_*, can be represented as:

(10) $$\begin{array}{ccc} \omega_{\mathrm{qua},x}=\pm \sqrt{\frac{\left(+a+b-c\right)}{2}} & \omega_{\mathrm{qua},y}=\pm \sqrt{\frac{\left(+a-b+c\right)}{2}} & \omega_{\mathrm{qua},z}=\pm \sqrt{\frac{\left(-a+b+c\right)}{2}} \end{array}$$

which indicates that there are eight possible combinations because each scalar angular velocity has two possible solutions. When one or more values of the quadratic sums, *a*, *b*, and *c*, are very close to zero, the estimated angular velocity should be around zero, so the sign ambiguity problem vanishes and the number of combinations declines. However, in general situations when eight candidates appear, the selection process is required. Intuitively, the derived and readily-available angular acceleration shown in Equation (7) can be involved in this process. Without loss of generality, assuming **ω***_j−1_* is the correctly estimated angular velocity at time stamp *j − 1*, the intuitive method to derive correct **ω***_j_* is to obtain the initial guess of **ω***_j_* by integration method shown in Equation (8), **ω***_int,j_*, and this guess is utilized as the reference to select one correct answer out of the eight candidates shown in Equation (10). More specifically, the L_2_ norm can be utilized as the quantitative measure:

(11) $${\left\|\omega_{\mathrm{qua}}-\omega_{\mathrm{int}}\right\|}_{2}$$

where the candidate with minimum value represents the correct choice. Figure 3(a) roughly sketches this computation process.

Practically, the quadratic method described above is likely to select an incorrect answer while the magnitude of the actual angular velocity, **ω***_act_*, is small. If the derived **ω***_j−1_* at time stamp *j* − *1* is precise, the most likely cause of estimation error of **ω***_j_* at time stamp *j* “in the perfect world” is the process of trapezoid integration, which assumes the acceleration is constant during that time interval. As depicted in Figure 3(a), the actual motion pattern may vary in a very fast manner, and the angular acceleration derived from the 12-axis GF-IMU catches the instant value at the sampled time stamp because of the memoryless computation shown in Equation (7). The discrepancy between the instant and average accelerations during time stamp *j* results in the estimation error of the initial-guess, **ω***_int,j_*. This phenomenon in the traditional IMU (TIMU) which derives the angular acceleration by the differentiation of the angular velocity signal is even worse since the differentiation process introduces the noise and delay as shown in Figure 3(a). In the empirical world the situation is even more severe due to signal noises and accumulated digitization round-off errors during computation. For example, the estimated **ω***_j−1_* at the *j* − *1* time stamp may already have certain estimation error, and the calculated angular acceleration and quadratic angular velocity shown in Equation (7) also have certain errors due to digitized matrix inversion and noisy sensor signals as depicted in Figure 3(b). Both empirical effects strongly affect the accuracy of 1-out-of-8 selection process, especially when the magnitude of the actual angular velocity **ω***_act_* is small, as depicted in Figure 3(c) where multiple choices of **ω***_qua_* may fall into the estimated ranges. In addition, because the estimation process is iterative, one incorrect estimate may badly affect the correctness of future estimates. Therefore it can be concluded that the quadratic method is suitable while the magnitude of quadratic sums, *a*, *b*, and *c*, are either very close to zero or large (*i.e.*, not small).

In summary, neither one of the two methods is individually capable of yielding a correct estimation of the angular velocity. Because of the complementary characteristics between them, it is intuitive to fuse signals from these two methods to yield a better angular velocity estimate, **ω***_12-axis_*.

### 3.2. Context-Based Interacting Multiple Models

A better estimation of the angular velocity can be achieved by the adequate combination of the signals derived from the integration and quadratic methods. The process can be categorized within the domain of Interacting Multiple Models (IMM) [27,28], which generally calculates the accuracy of all models in a stochastic manner and mixes the estimated signals from all sources in a weighted manner to produce the correct estimate. Because executing the covariance of all models requires certain computation power as well as the performance of the models for specific scenarios may not be fairly judged by simple Gaussian assumptions, the context-based IMM [26] is adopted in the developed algorithm, which introduces the pre-selected contexts as the basic judgment for signal mixture from multiple models.

**Figure 4.**
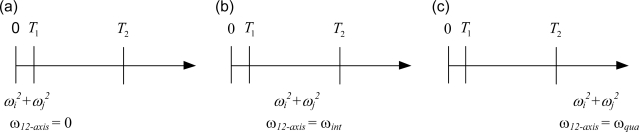
Three different computation methods categorized by the magnitude of the quadratic terms: (**a**) 0 ≤ *ω_i_^2^* + *ω_j_^2^* ≤ *T_1_*; (**b**) *T_1_* ≤ *ω_i_^2^* + *ω_j_^2^* ≤ *T_2_*; (**c**) *ω_i_^2^ + ω_j_^2^ ≥ T_2_*.

The development shown in the previous sub-section indicates that the quadratic method is effective while the magnitude of the angular velocity is either close to zero or very large. Therefore, two thresholds, *T_1_* and *T_2_*, are selected as the contexts. *T_1_* is the boundary where the estimated angular velocity should be treated as zero, and *T_2_* is the boundary where the quadratic method is effective. These two contexts divide the range of quadratic sums, *a*, *b*, and *c*, into three sections as depicted in Figure 4. While 0 ≤ *ω_i_^2^* + *ω_j_^2^* ≤ *T_1_* as shown in Figure 4(a), referred to as the zero model, one can set the angular velocity to zero. While *ω_i_^2^* + *ω_j_^2^* ≥ *T_2_* as shown in Figure 4(c), referred to as the quadratic model, one can obtain the angular velocity by the quadratic method. While *T_1_* ≤ *ω_i_^2^* + *ω_j_^2^* ≤ *T_2_* as shown in Figure 4(b), referred to as the integration model, one adopts the integration method.

Each of the quadratic sums, *a*, *b*, or *c*, can reside in three possible sections shown in Figure 4, so there are twenty-seven possible combinations. Because the equations shown in Equation (9) are coupled, further categorization and treatment is detailed as follows.

#### **Case 1.** *a, b, c ≥ T_2_*

**Figure 5.**
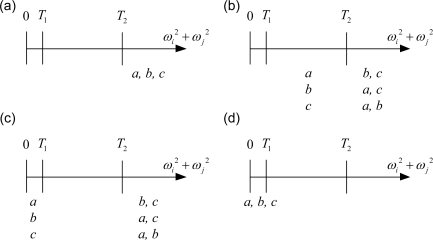
Four different scenarios which utilize different algorithms.

When *a, b, c* ≥ *T_2_* as shown in Figure 5(a), **ω***_act_* is far away from zero along all three principal directions. In this case the estimated **ω***_12-axis_* is determined by the quadratic method:

(12) $$\begin{array}{ccc} \omega_{12–\mathrm{axis},x}=\omega_{\mathrm{qua},x} & \omega_{12–\mathrm{axis},y}=\omega_{\mathrm{qua},y} & \omega_{12–\mathrm{axis},z}=\omega_{\mathrm{qua},z} \end{array}$$

#### **Case 2.** *T_1_ ≤ a < T_2_* and *b, c ≥ T_2_*

When *T_1_ ≤ a < T_2_* and *b, c ≥ T_2_* as shown in Figure 5(b), it is reasonable to conclude that **ω***_act,z_* is far away from zero and **ω***_act,x_* and **ω***_act,y_* are likely to have moderate magnitudes. Therefore both methods are utilized in this case:

(13) $$\begin{array}{ccc} \omega_{12–\mathrm{axis},x}=\omega_{\mathrm{int},x} & \omega_{12–\mathrm{axis},y}=\omega_{\mathrm{int},y} & \omega_{12–\mathrm{axis},z}=\omega_{\mathrm{qua},z} \end{array}$$

Similarly, both *T_1_ ≤ b < T_2_*, *a, c ≥ T_2_* and *T_1_ ≤ c < T_2_*, *a*, *b ≥ T_2_* are within this case.

#### **Case 3.** *0 ≤ a < T_1_* and *b, c ≥ T_2_*

When *0 ≤ a < T_1_* and *b, c ≥ T_2_* as shown in Figure 5(c), it can be concluded that **ω***_act,z_* has a large magnitude but **ω***_act,x_* and **ω***_act,y_* are close to zero. Therefore only the former requires computation by the quadratic method:

(14) $$\begin{array}{ccc} \omega_{12–\mathrm{axis},x}=0 & \omega_{12–\mathrm{axis},y}=0 & \omega_{12–\mathrm{axis},z}=\omega_{\mathrm{qua},z} \end{array}$$

Similarly, both *0* ≤ *b < T_1_*, *a, c ≥ T_2_* and *0* ≤ *c < T_1_*, *a, b ≥ T_2_* are within this case.

#### **Case 4.** *0 ≤ a, b, c < T_1_*

When *0* ≤ *a, b, c < T_1_* as shown in Figure 5(d), it is reasonable to set all components to zero:

(15) $$\begin{array}{ccc} \omega_{12–\mathrm{axis},x}=0 & \omega_{12–\mathrm{axis},y}=0 & \omega_{12–\mathrm{axis},z}=0 \end{array}$$

Besides the four cases shown above, there are nineteen combinations left undetermined. Because there is no clear trend to judge the adequateness of the quadratic method in these combinations, the estimated angular velocity **ω***_12-axis_* is derived from the integration method (*i.e.*, **ω***_12-axis_* = **ω***_int_*). If the computed *a*, *b*, or *c* appears in an unreasonable less-than-zero value due to empirical computation error, the estimated angular velocity **ω***_12-axis_* is also derived from the integration method (*i.e.*, **ω***_12-axis_* = **ω***_int_*, the quadratic sums are not utilized in the computation in this time stamp).

**Figure 6.**
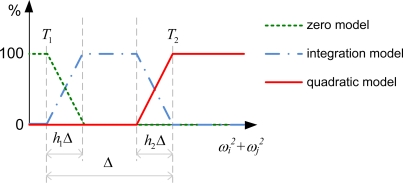
Defined probabilities of the interacted three models.

The proposed estimation shown in the previous paragraph has the “hard switching” nature. This implies an unreasonable situation because the trustworthiness of the models has a sharp boundary. Technically, suddenly switching the estimation from one method to another also introduces a signal discontinuity problem. Therefore the “soft switching” technique is adopted, which defines the probability of each model in a continuous manner as shown in Figure 6. When ω*_i_^2^* + ω*_j_^2^* is close to *T_1_*, **ω***_12-axis_* is designed to be the linear combination of zero and **ω***_int_* :

(16) $$\omega_{12–\mathrm{axis}}=(1-\frac{\omega_{i}^{2}+\omega_{j}^{2}-T_{1}}{h_{1}\Delta })0+(\frac{\omega_{i}^{2}+\omega_{j}^{2}-T_{1}}{h_{1}\Delta })\omega_{\mathrm{int}}$$

where Δ = *T_2_* − *T_1_*. When *ω_i_^2^* + *ω_j_^2^* is close to *T_2_*, **ω***_12-axis_* is designed to be the linear combination of **ω***_int_* and **ω***_qua_* :

(17) $$\omega_{12–\mathrm{axis}}=(\frac{T_{2}-(\omega_{i}^{2}+\omega_{j}^{2})}{h_{2}\Delta })\omega_{\mathrm{int}}+(\frac{\left(\omega_{i}^{2}+\omega_{j}^{2})-(T_{2}-h_{2}\Delta \right)}{h_{2}\Delta })\omega_{\mathrm{qua}}$$

The *h_1_* and *h_2_* are the percentages of the overall range to be utilized for linear combination of models (*i.e.*, soft switching). Smaller values of *h_1_* and *h_2_* represent sharper switching, and larger values represent smoother transition. The *h_1_* and *h_2_* are set around 2% in the empirical evaluation.

### 3.3. Brief Discussion

The algorithm reported in the previous sub-section utilizes the first three terms shown in Equation (3) to recover the angular velocity. The last three terms can also be utilized to perform the quadratic inversion. Assuming the quadratic multiplication is labeled as:

(18) $$\begin{array}{ccc} \omega_{x}\omega_{y}=d & \omega_{x}\omega_{z}=e & \omega_{y}\omega_{z}=f \end{array}$$

and the angular velocity can be derived as:

(19) $$\begin{array}{ccc} \omega_{x}=\pm \sqrt{\frac{\mathrm{de}}{f}} & \omega_{y}=\pm \sqrt{\frac{\mathrm{df}}{e}} & \omega_{z}=\pm \sqrt{\frac{\mathrm{ef}}{d}} \end{array}$$

Because of the multiplication characteristics there are only two candidates instead of eight. This reveals that if one sign of ω*_i, i=x,y,z_* is selected, the other two will be determined. However, empirically the sign determination is tricky and no obvious model can be constructed. Thus this approach is not adopted in the development. Another method is to construct the complete squares by utilizing all six terms shown in Equation (3):

(20) $$\begin{array}{cccccc} \omega_{x}^{2}+\omega_{y}^{2}=a & \omega_{x}^{2}+\omega_{z}^{2}=b & \omega_{y}^{2}+\omega_{z}^{2}=c & \omega_{x}\omega_{y}=d & \omega_{x}\omega_{z}=e & \omega_{y}\omega_{z}=f \end{array}$$

and angular velocity can be derived as:

(21) $$\begin{array}{l} \omega_{1}+\omega_{2}=\pm \sqrt{{\left(\omega_{1}+\omega_{2}\right)}^{2}}=\pm \sqrt{\left(a+2d\right)}\\ \omega_{1}+\omega_{3}=\pm \sqrt{{\left(\omega_{1}+\omega_{3}\right)}^{2}}=\pm \sqrt{\left(b+2e\right)}\\ \omega_{2}+\omega_{3}=\pm \sqrt{{\left(\omega_{2}+\omega_{3}\right)}^{2}}=\pm \sqrt{\left(c+2f\right)} \end{array}$$

Though the sensed signals shown in Equation (7) are utilized in a more thorough manner, the strategy of setting adequate contexts is also not clear in this case. For example, the advantage of the zero model does not exist in this case because the setting of *ω_x_* + *ω_y_* = *0* can only reveal that *ω_x_* and *ω_y_* are in opposite sign with no magnitude information. Thus this approach is not adopted in the development either.

## 4. Experimental Evaluation

The experimental apparatus shown in Figure 2(b) was utilized for experimental evaluation of the proposed 12-axis system. The required sensory measurements were obtained from four 3-axis accelerometers (ADXL330, ±3g, Analog Device) installed at the specific configuration shown in Figure 2(a). In addition, a traditional IMU composed by one 3-axis accelerometer (ADXL330) and three 1-axis rate gyros (ADXRS610, ±3000/s, Analog Device) was also mounted at the COM for performance comparison. A real-time embedded control system (sbRIO-9632, National Instruments) running at 500 Hz was in charge of sensor signal collection. All of the analog input channels of the sbRIO have ±10 V range and 16-bit A-to-D resolutions. Random motions with varied magnitudes were applied to the experimental apparatus during experiments and the following analysis was based on the measured data.

### 4.1. Selection of Contexts T_1_ and T_2_

The context *T_1_* represents the boundary which sets the estimated angular velocity **ω***_12-Axis_* at zero. It is not reasonable to set a large *T_1_* as it would force **ω***_12-Axis_* to be zero when it is not. On the other hand, a very small *T_1_* yields very little data that qualifies for this criterion. Empirically it is determined by the noise level of the sensors as well as the precision of the digitized computation. *T_1_* is set around 0.1 in the experiments.

The context *T_2_* determines the magnitude level where the angular velocity can be effectively determined by quadratic methods instead of the integration method. Therefore small *T_2_* easily yields the wrong selection from the eight candidates. Large *T_2_* forces the data to be computed by integration, and the data drift appears when the time duration of the angular velocity computed in this method is long. Therefore a study on how to correctly choose the right *T_2_* is performed and detailed as follows.

**Figure 7.**
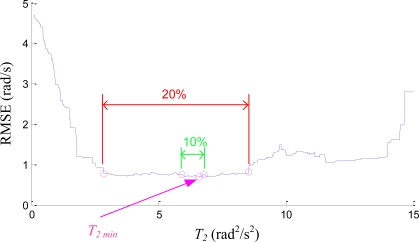
The relationship between *T_2_* and the RMSE between **ω***_TIMU_* and **ω***_12-Axis_*.

Figure 7 plots the typical Root-mean-squared Error (RMSE) *vs. T_2_* based on one of the experimental data, where the RMSE is the comparison between the estimated angular velocity, **ω***_12-Axis_*, and that measured by the traditional IMU, **ω***_TIMU_*. The RMSE shown in the plot is the summed result of its three scalar components. The plot indicates that the RMSE is relatively large when *T_2_* is small, when the quadratic method is over trusted. It also indicates that RMSE is relatively large when *T_2_* is large. In this setting most of the estimates were done by the integration method and the data drift was observed. The wide and flat bottom of the curve shown in Figure 7 is also observed in other data sets, which indicates that there exists a wide selectable range of *T_2_* values which yields similar performance, as the best RMSE happened at *T_2 min_*. For example, if the acceptable RMSE is bounded by an extra 20% of the best RMSE, the selectable range of *T_2_* is spanned from 3 to 9.

**Figure 8.**
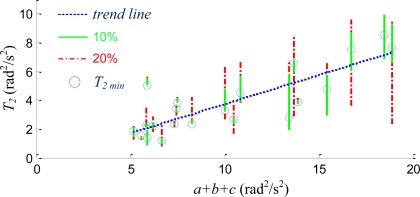
The relation between amplitude of motion and *T*_2min_.

Figure 8 plots the variation of *T_2 min_* (blue circle) *vs.* the average level of the motion, which is quantitatively defined as the summation of the quadratic sums, *a + b + c*. Instead of defining the level of motion directly in the angular velocity, quadratic sums are utilized since these sums are available right after the multiplication of the inversed matrix to the sensory inputs shown in Equation (7). Because the errors resulted from the matrix inversion and noises due to empirical sensor readings are usually scaled with the magnitude of the signals, the selected *T_2_* should increase as the magnitude of motion increases. The blue linear trend line with positive slope also confirms this phenomenon. The plot also reveals that the tolerable 10% or 20% increase of RMSE intersects with the linear trend line. Because the lengths of 20% lines are large and the slope of the trend line is small, the computation error of *a + b + c* has very little effect on the quantitative measure of the trend line. Thus the adequate *T_2_* can be easily obtained according to the equation of the trend line when the quadratic sums, *a*, *b*, and *c*, are given. This plot suggests that the selection of *T_2_* can be achieved by the given quadratic sums and the trend line, and the RMSE comparison test which requires the gyroscope input shown in Figure 7 is not necessary. The selected *T_2_* is fixed for the followed real-time estimation.

**Figure 9.**
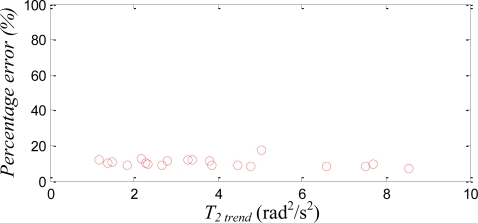
The relationship between *T_2_* and the ratio of RMSE and amplitude of angular velocity.

In order to quantitatively evaluate the usability of the trend line, instead of using *T_2 min_* as the context, Figure 9 plots the percentage error of the estimated angular velocity *versus T_2 trend_*, which is the selected *T_2_* calculated from the trend line with given quadratic sums. Percentage error is calculated as the ratio of the RMSE to the maximum magnitude at that experiment trial, where the RMSE is the comparison between the estimated angular velocity, **ω***_12-Axis_*, and that measured by the traditional IMU, **ω***_TIMU_*. Figure 9 indicates that the computed *T_2_* from trend line performs adequately; the percentage errors are mostly around 12% or less.

### 4.2. Performance of the State Derivation from the 12-Axis GF-IMU

**Figure 10.**
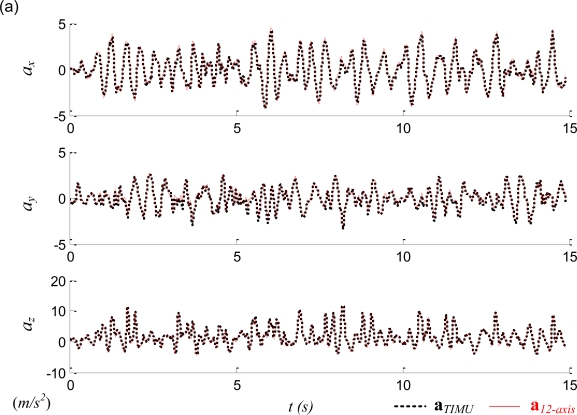

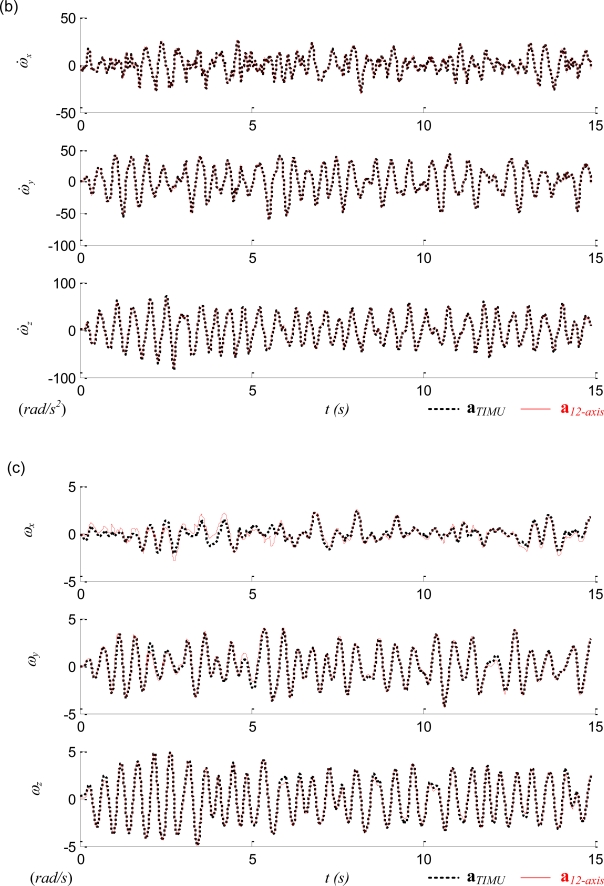
Comparison of states obtained from the traditional IMU (thick black dashed line) and the 12-axis IMU (thin red solid line): (**a**) linear acceleration, (**b**) angular acceleration, and (**c**) angular velocity.

In the experimental evaluation the apparatus was moved arbitrarily in the 3-dimensional space; thus the linear and angular accelerations along all three principal axes could be induced in the test. Before the sensor readings were imported into Equation (5), the raw accelerometer readings were filtered by Chebyshev filters and gravity-compensated by the readings of the 2-axis inclinometer. Table 1 lists the statistical summary of the experiments where the RMSEs were the comparisons between the estimated state of **ω***_12-Axis_* and the measured state from the traditional IMU installed at the COM, **ω***_TIMU_*. The angular acceleration of the traditional IMU is obtained by differentiation of the angular velocity, followed by filtered with a Chebyshev filter. Figure 10 plots one typical result of the experiment. Figure 10(a) confirms that though the sensors of the proposed 12-axis GF-IMU (thin red solid lines) are not located at the COM, they can indeed recover the COM linear acceleration. Figure 10(b) shows that the angular acceleration can also be correctly derived by the 12-axis GF-IMU. Figure 10(c) reveals that though several unmatched sections exist between the 12-axis GF-IMU and the traditional IMU readings, the proposed algorithm in general can indeed recover the angular velocity along with all three principal directions. The discrepancy either resulted from (i) the accumulated integration error where the magnitude of the quadratic sums fell into the integration model for too long or (ii) the incorrect selection of the angular velocity in the quadratic model.

**Figure 11.**
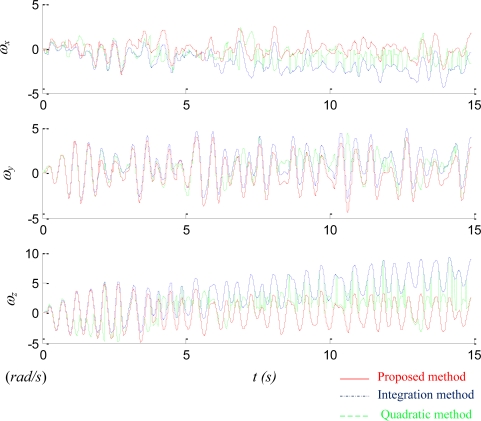
Comparison of the angular velocity derived from three different methods: integration method (blue dash-dotted line)), quadratic method (green dashed line), and proposed method (red solid line).

Figure 11 compares the performance of three different methods: integration method, quadratic method, and the proposed method. Figure 10(c) reveals that the angular velocity derived from the proposed method matches closely to the readings from the traditional IMU, so in Figure 11 the performance of the latter one is skipped for clear presentation. Figure 11 shows that the angular velocity derived from the integration method drifts over time as expected. In contrast, the angular velocity derived from the quadratic method is bounded. However, the 1-out-of-8 selection process of the quadratic method is likely to select an incorrect answer while the magnitude of the signal is small. In addition, because the estimation process is iterative, one incorrect estimate may badly affect the correctness of future estimates until at certain moment the correct selection moves the estimates back to the right track. Figure 11 indicates that the proposed method with right mixture between the integration and the quadratic methods yields the adequate estimation.

**Figure 12.**
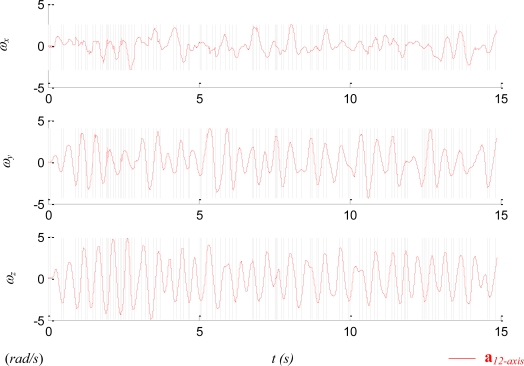
The timing of switching (grey lines) between the integration method and the quadratic methods.

Figure 12 shows the timings where the switching between two methods takes place. The 15-sec data had 7,500 sampled data points, and the proposed method switched around 100 times.

## 5. Conclusions

We have investigated a 12-axis inertial measurement unit that utilizes four 3-axis linear acceleration measurements from accelerometers installed at four distinct locations. We have developed a new algorithm which derives the angular velocity by mixing the signals from its quadratic form and its derivative form via the context-based interacting multiple models. The performance of the system was evaluated while the system was under arbitrary 3-dimensional motion. By adequately-choosing two contexts, the angular velocity can indeed be recovered. In the meantime, the linear and angular accelerations are correctly estimated as well, which confirmed that the COM acceleration state can be derived even though the sensors are not installed at that specific spot.

We are in the process of investigating a sensor fusion scheme of the reported system with other position and orientation sensors with the intention of constructing an observable system capable of accurate full body state estimation for analysis of dynamic locomotion in legged robots.

## Figures and Tables

**Table 1.** The RMSE Between the traditional IMU and the 12-axis IMU while the apparatus moved arbitrarily in the 3-dimensional space.

**Linear acceleration (m/s^2^)**			**Angular acceleration (rad/s^2^)**			**Angular velocity (rad/s)**		
*a_x_*	*a_y_*	*a_z_*	*ω̇_x_*	*ω̇_y_*	*ω̇_z_*	ω*_x_*	ω*_y_*	ω*_z_*
0.1359	0.0933	0.1296	0.4985	0.7691	1.4188	0.3953	0.2301	0.2593
